# CBCT-Based Analysis of Medial and Lateral Pterygoid Plates: Cross-Sectional Study of Saudi Subpopulation

**DOI:** 10.3390/jcm15030951

**Published:** 2026-01-24

**Authors:** Zuhair Alkahtani, Hassan Ahmed Assiri, Mohammad Hassan Alasiri, Waleed A. Asiri, Hashim Fayez Alshehri, Abdulrahman N. Almubarak, Raed K. Alqahtani, Ali Azhar Dawasaz, Sonia Egido-Moreno, José López-López

**Affiliations:** 1 Department of Pediatric Dentistry and Orthodontic Sciences, College of Dentistry, King Khalid University, Abha 62521, Saudi Arabia; zmalqhtany@kku.edu.sa; 2 Department of Diagnostic Sciences and Oral Biology and Periodontology, College of Dentistry, King Khalid University, Abha 62529, Saudi Arabia; 3 Head of the Department of Oral and Maxillofacial Radiology, Dental Hospital, College of Dentistry, King Khalid University, Abha 62529, Saudi Arabia; 4 Internship Program, College of Dentistry, King Khalid University, Abha 62529, Saudi Arabiawasiry19@gmail.com (W.A.A.); hfmfshehri@gmail.com (H.F.A.);; 5 Department of Dental Research, D.Y. Patil Dental College & Hospital, D.Y. Patil Vidyapeeth (Deemed to be University), Pune 411018, India; 6 Service of the Medical-Surgical Area of Dentistry Hospital, Faculty of Dentistry, University of Barcelona, 08007 Barcelona, Spain18575jll@gmail.com (J.L.-L.); 7 Department of Odontostomatology, Faculty of Dentistry, University of Barcelona, 08907 Barcelona, Spain

**Keywords:** cone beam computed tomography, pterygoid process, medial pterygoid plate, lateral pterygoid plate, morphometry, pterygoid implants, posterior maxillary

## Abstract

**Background**: The pterygoid plates serve as crucial reference points for posterior maxillary surgery and the placement of pterygoid implants; however, population-specific morphometric reference values remain underexplored for adults of Asir region (Abha city) of Saudi Arabia. **Methods**: This retrospective cross-sectional cone beam computed tomography (CBCT) study analyzed the archived scans obtained at King Khalid University Dental Hospital. Of 100 randomly selected adult CBCT scans collected between June and October 2025, 50 images met the eligibility criteria. The analyses were conducted using OnDemand3D software to measure the bilateral pterygoid plates’ length, thickness at the maximum diameter, and medial-lateral divergence angle. Styloid process length was measured as an exploratory variable. Three calibrated examiners performed the measurements, and the reliability was assessed using interclass correlation coefficients. **Results**: Fifty CBCT scans met the inclusion criteria (30 males, 20 females). The mean lateral pterygoid plate length was 14.61 ± 3.69 mm on the right and 13.83 ± 3.93 mm on the left, while the mean medial plate length was 11.27 ± 3.52 mm (right) and 11.98 ± 3.82 mm (left). Side to side paired comparisons showed no significant right–left differences in lateral plate length (mean R–L 0.79 mm, 95% CI −0.48 to 2.06), lateral thickness (mean 0.04 mm, 95% CI −0.14 to 0.22), medial thickness (mean 0.01 mm, 95% CI −0.19 to 0.21), or pterygoid angulation (mean 1.99°, 95% CI −1.07 to 5.05), supporting bilateral symmetry. Bilateral correlations were strong for medial plate length (r = 0.729, *p* < 0.001) and angulation (r = 0.632, *p* < 0.001). Males had a longer right lateral plate than females (15.74 ± 3.55 mm vs. 12.93 ± 3.31 mm; mean difference 2.81 mm, 95% CI 0.80–4.82; *p* = 0.007), whereas other measurements did not differ by sex. Plate thickness ranged from approximately 1.33 to 1.46 mm and left medial plate thickness correlated negatively with left medial plate length (r = −0.399, *p* = 0.004). Styloid process length averaged 22.99 ± 9.76 mm and showed no significant association with pterygoid plate measures. **Conclusions**: CBCT-derived findings demonstrated overall bilateral symmetry and limited dimorphism in relation to sex. These region-specific morphometries support individualized preoperative posterior maxillary surgery and pterygoid implant planning.

## 1. Introduction

The pterygoid process of the sphenoid is characterized by the presence of paired medial and lateral pterygoid plates. These structures are integral to the posterior nasal and infratemporal regions, serving as the osseous origins for the medial and lateral pterygoid muscles. These muscles play a key role in mastication and mandibular kinematics, while the plates define the posterior boundary of the pterygopalatine and pterygomaxillary regions, which are pathways for major neurovascular structures. Due to their anatomical and functional centrality, even subtle variations in pterygoid plate morphology may influence oral and maxillofacial surgery, orthodontic treatment planning, and implant rehabilitation [1,2]. In numerous maxillofacial operations, the pterygomaxillary junction (PMJ) and pterygoid plates are significant anatomical sites. In the context of Le Fort I osteotomy, the surgical approach and angulation of pterygomaxillary separation can impact the risk of unfavorable pterygoid plate fractures and hemorrhage; thus, preoperative cross-sectional imaging of the PMJ is essential for enhanced surgical planning and safer disjunction [3]. Cone beam computed tomography (CBCT) is an advanced three-dimensional imaging method commonly used in the maxillofacial region. However, challenges such as elevated radiation doses, high costs, limited availability, prolonged scanning durations, suboptimal resolution, and interpretation challenges have constrained the application of computed tomography (CT) in dentistry. Some of these issues can be addressed using CBCT, which offers several advantages over traditional CT in oral and maxillofacial imaging [4]. Thus, CBCT emerges as a reliable tool for examining the pterygomaxillary region, including the assessment of the pterygoid plates, particularly when a large field of view is applied [5,6]. Overall, evidence-based practice guidelines for CBCT should be clinically justified and optimized, with the smallest field of view and lowest exposure settings that are still appropriate for the diagnostic task, in line with ALARA radiation protection principles [7]. According to studies using CBCT and multidetector CT (MDCT), the choice of technique and regional bone sizes around the PMJ predict fracture patterns and safety margins for osteotomy [8]. However, outside of orthognathic surgical procedures, the success of pterygoid implants depends on the engagement of the pterygoid process to rehabilitate the atrophic posterior maxilla. CBCT-based studies have described possible implant angulations and lengths, as well as three-dimensional variations of the pterygomaxillary complex and the pterygomaxillary implant [9]. For the evaluation of hard tissue within the craniofacial skeleton, CBCT offers excellent spatial resolution and submillimeter linear accuracy, rendering it suitable for quantitative morphometry of delicate plates and fissures. Systematic reviews indicate that CBCT yields reliable linear measurements for implant planning, with errors typically under 1 mm for applicable voxel sizes [10]. The effective doses associated with CBCT are typically lower than those of MDCT within the same maxillofacial fields of view, thus establishing CBCT as the preferred modality when three-dimensional detail is required and conventional radiography is inadequate, if justification and optimization follow evidence-based guidelines [11]. Despite the clinical significance of this anatomy, normative CBCT data for the medial and lateral pterygoid plates remain limited. Almost all reported morphometric work has been restricted to nearby structures such as the PMJ or pterygomaxillary fissure and usually involve small or geographically restricted samples [12]. Recent studies utilizing CBCT have begun to measure pterygoid-related dimensions, divergence angles, and separation patterns, focusing on variations by side, sex, skeletal pattern, and dental status [2]. The interpretation of pterygoid-region morphometry also relies on age and population background. Therefore, CBCT studies of the pterygomaxillary region performed for surgical risk assessment have revealed that thickness-related parameters may vary by sex, suggesting demographic factors could determine bony morphology in this anatomic corridor [6]. Furthermore, CBCT-based morphometry of pterygoid-process substructures such as the pterygoid hamulus demonstrates measurable dimensional changes with age, providing evidence that it is more prudent to assess age as a potential correlate of pterygoid-region anatomy than assume stability during adulthood [13]. Lastly, craniofacial norms differ by population; Saudi-specific cephalometric datasets, unlike widely utilized European-American references, provide strong support for region-specific reference values for craniofacial planning [14].

Nevertheless, there exists a scarcity of population-specific datasets pertaining to Saudi Arabia. A recent study in Saudi Arabia has demonstrated the feasibility of pterygoid plate morphometry through CBCT in local patients; however, comprehensive, region-specific reference data remain scarce [15]. Establishing population-appropriate normative values is a prerequisite for making precise diagnoses and performing surgery safely. Numerous studies have indicated that craniofacial norms among Saudi and other Middle Eastern populations differ from Euro-American standards and may exhibit regional variations within the Kingdom, emphasizing the limitations of uncritically adopting external datasets [16]. Abha, the capital of the Asir Region located in southwestern Saudi Arabia, hosts a sizeable highland population characterized by distinctive demographic and eco-geographic characteristics. Local centres in Abha routinely perform orthognathic, implant, and orthodontic procedures where the morphology of the pterygoid plate may influence the risk of PMJ separation fractures and the design of pterygoid implants or posterior maxillary reconstructions. Accurate CBCT-based morphometry of the pterygoid plates has the potential to directly improve preoperative planning and risk stratification for this population. Pertaining to this specific population-targeted study, data regarding plate thickness (relevant to fracture susceptibility and surgical manipulation), divergence angulation (concerning surgical trajectories), and bilateral agreement metrics remains insufficient. Furthermore, methodologies and landmark definitions vary significantly among studies, complicating the comparison of results across groups. The length of the styloid process was included as an exploratory craniofacial feature to evaluate if variations in the stylohyoid complex correspond with the morphology of the pterygoid plate in this population. Therefore, we aimed to perform CBCT analysis to quantify key morphometric parameters of the medial and lateral pterygoid plates including length, thickness, divergence angulation, and styloid process length in adult residents of Abha and evaluate differences according to sex and side. By generating region-specific normative data, the study seeks to address a recognized evidence gap and to inform orthognathic planning for pterygomaxillary separation, pterygoid implant placement, and other maxillofacial interventions in this population.

## 2. Materials and Methods

### 2.1. Study Design and Setting

A retrospective cross-sectional CBCT study was conducted using existing scans from adult patients who attended King Khalid University, College of Dentistry (KKUCOD), Abha, Saudi Arabia. The CBCT images were randomly selected between June to October 2025, representing a cohort of residents from Abha city in the Asir region of Saudi Arabia. The study was approved by the Research Ethics Committee at King Khalid University (HAPO-06-B-001) (KKU-123-2025-31). The research was conducted in compliance with the Declaration of Helsinki [17]. Patients who sought treatment at the hospital signed a consent form allowing their data to be used for educational or research purposes.

### 2.2. Sample

Archived scans were randomly selected from patients who had previously undergone CBCT imaging for various therapeutic purposes, such as orthognathic surgery and pathology delineation. A computer-generated random-number sequence was employed to ensure an unbiased selection process. The study included CBCT examinations of 100 adults (≥20 years) of both sexes, all of whom were residents in Abha at the time of imaging. A total of 50 scans of the patients met the eligibility criteria and further analyzed. Regarding sample calculation and power analysis, this study is a retrospective, cross-sectional analysis of CBCT morphometrics utilizing archived clinically indicated scans. Consequently, no formal a priori power calculation was conducted, nor was a pilot study dataset utilized to determine sample size since the analytical sample comprises the number of randomly selected scans over the study period that were eligible and satisfied the specified image-quality requirements. To provide quantitative interpretation, we evaluated estimation precision based on the observed variability in our dataset (*n* = 50; males *n* = 30; females *n* = 20) and conducted a sensitivity analysis to determine the “minimal detectable effect.” The estimated half-width of the 95% confidence interval around the mean for the most pertinent morphometric measures is approximately ±1.05 mm for right lateral pterygoid length (SD 3.69 mm) and ±0.18 mm for right medial pterygoid thickness (SD 0.64 mm), facilitating clinically interpretable precision in the reporting of reference values. The sample provides approximately 80% power for sex-based comparisons (30 vs. 20, two-sided α = 0.05) and suggests a moderate-to-large standardized difference (Cohen’s d ≈ 0.83), with an absolute mean difference of approximately 2.85 mm for right lateral pterygoid length (pooled SD ≈ 3.46 mm) and approximately 0.53 mm for right medial pterygoid thickness (pooled SD ≈ 0.65 mm). In paired right-left comparisons (*n* = 50, two-sided α = 0.05), approximately 80% power corresponds to a standardized paired effect size of dz ≈ 0.40, indicating a mean right-left difference of about 1.80 mm for lateral plate length (SD of paired differences 4.46 mm) and 0.26–0.29 mm for plate thickness (SD of paired differences 0.65–0.71 mm).

### 2.3. Eligibility Criteria

The inclusion criteria specified that the CBCT scans must encompass the posterior maxilla and sphenoid region, possess sufficient image quality for the assessment of the pterygoid plate, be free from artifacts, and have no history of maxillofacial trauma or orthognathic surgery.

### 2.4. Imaging Protocol

All CBCT scans were acquired using KaVo Dental GmbH (Biberach, Germany). Measurements were conducted using OnDemand3D^®^ software version 1.0 (Build 1.0.10.7462) (×64 Edition) (Cybermed Inc., Seoul, Republic of Korea). The specified field of view was 8 × 15 cm, with acquisition settings (90 kVp, 6.3 mA, and 4.5 s), and a voxel size of 0.2 mm. The scans were reconstructed with an isotropic voxel size appropriate for linear craniofacial measurements (0.2 mm) and standard clinical exposure settings. The voxel size and field of view were selected to visualize the sphenoid and pterygoid processes while adhering to the optimization principles for CBCT examinations. These parameters fall within the validated ranges for accurate linear CBCT measurements of maxillofacial structures. Digital Imaging and Communications in Medicine (DICOM) datasets were imported into OnDemand3D^®^ software version 1.0 (Build 1.0.10.7462) (×64 Edition), and multiplanar reconstruction (axial, coronal, sagittal) was used to standardize head orientation, utilizing the midsagittal plane and palatal plane as references, following CBCT protocols established in previous studies of the pterygoid region.

### 2.5. Measurements

**Figure 1:**
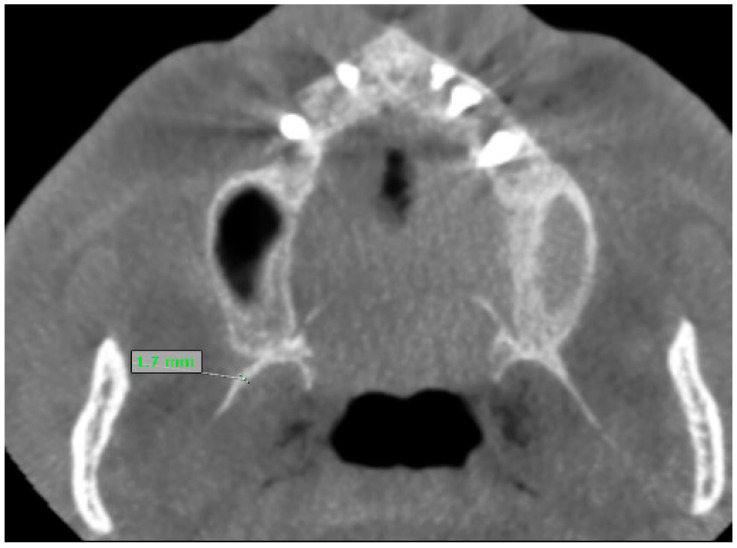
Illustration revealing the way the thickness of plate was measured on CBCT axial plane.

**Figure 2:**
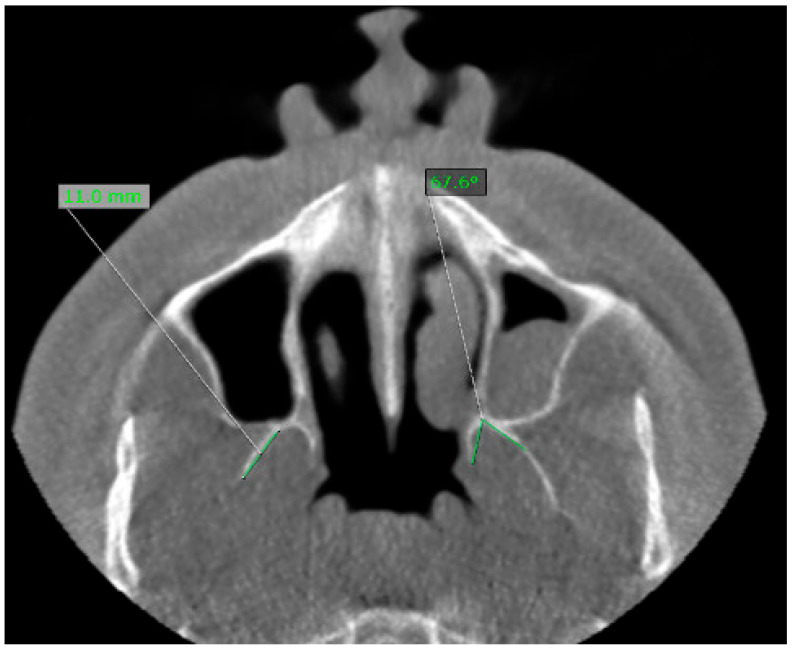
Illustration revealing the way the length and the divergence angle were measured on CBCT axial plane.

Bilateral linear and angular measurements were performed as follows:

- Lateral pterygoid plate length (RLP, LLP): the distance from the most distal point of the lateral pterygoid plate to the point of origin in the axial plane (Figure 1).
- Lateral pterygoid plate thickness (RLP, LLP): the thickness of the plate at its greatest diameter.
- Medial pterygoid plate length (RMP, LMP): the linear distance from the most distal or inferior tip of the medial plate to the origin.
- Medial pterygoid plate thickness (RMP, LMP): the thickness of the plate at its greatest diameter.
- Divergence angle: the divergence angle between the medial and lateral pterygoid plates, measured in the axial plane. For angular measurements, lines were constructed along plate long axis up to the midpoint of the distance between the medial and lateral pterygoid plates, and angles were computed automatically by the software (Figure 2).
- Styloid process length: the distance from the base of the styloid process at the temporal bone to its tip, measured on reformatted sagittal/oblique planes, using thresholds for elongation (≥30 mm).

### 2.6. Calibration and Reliability

For the calibration and reliability purposes, two examiners independently performed all measurements after the calibration session. Then, a random subset of 8/50 (16%) of CBCT was re-measured after a one-month interval to calculate the inter-examiner reliability for continuous intraclass correlation coefficients (ICC). The overall ICC for inter-examiner reliability was 0.82, indicating good agreement.

### 2.7. Statistical Analysis

Data were analyzed using Statistical Package for the Social Sciences (SPSS) version 29 (IBM Corp., Armonk, NY, USA). The normality of continuous variables was assessed using the Shapiro–Wilk test. Descriptive statistics (mean, standard deviation) were calculated for each variable in the total sample and stratified by sex. The appropriate tests are used as follows:

- Independent samples *t* tests were used to compare male and female groups for each measurement (equal variance assumed if Levene’s test *p* > 0.05).
- Paired *t* tests were used to evaluate side differences (right vs. left) for lengths, thicknesses, and angles.
- Pearson correlation coefficients were computed to explore associations between measurements (e.g., right vs. left sides, length vs. thickness, plate dimensions vs. styloid length). Then, the significance level was established at *p* < 0.05. Along with *p*-values, mean differences were given with 95% confidence intervals, and Hedge’s g was calculated for comparisons between sex. In case there are several morphometric comparisons, the results were looked at with an eye toward multiplicity, and clinically important effect sizes were highlighted along with statistical significance. In addition, Pearson correlation was employed to identify relationships between age and each morphometric characteristic. Additionally, sex-adjusted linear regression models were employed for each morphometric outcome, using age (in 10-year increments) and sex as predictors. We established the threshold for statistical significance at *p* < 0.05.

## 3. Results

### 3.1. Descriptive Statistics

**Figure 3:**
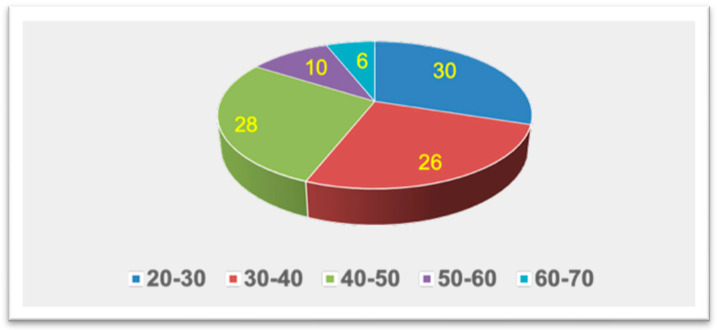
Age-wise frequency of the sample distribution.

**Figure 4:**
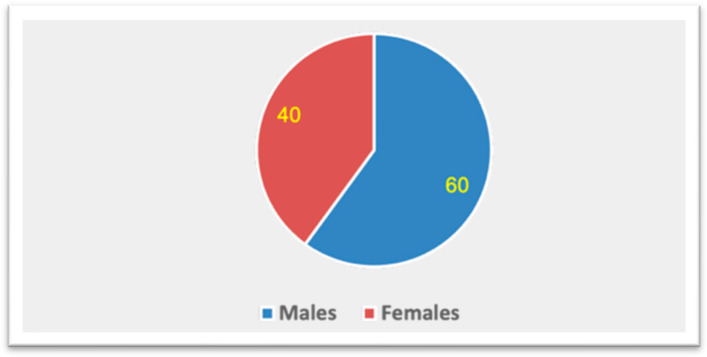
Sex-wise distribution of the sample.

The final analytic sample comprised 50 adults, including 30 (60%) males and 20 (40%) females. According to the grouped age distribution, participants were approximately 20–70 years old with an estimated mean age of 38.6 ± 11.9 years. The distribution of the sample in relation to age and sex is presented in Figure 3 and Figure 4. A summary of the morphometric measurements of the pterygoid plates and styloid process for the total sample categorized by sex (Table 1). Overall, the lateral pterygoid plates were longer than the medial pterygoid plates on both sides. The right lateral pterygoid plate length averaged 14.61 ± 3.69 mm (males: 15.74 ± 3.55 mm; females: 12.93 ± 3.31 mm), while the left lateral pterygoid plate length averaged 13.83 ± 3.93 mm (males: 13.62 ± 3.96 mm; females: 14.14 ± 3.99 mm). In comparison, the right medial pterygoid plate length averaged 11.27 ± 3.52 mm, and the left medial pterygoid plate length averaged 11.98 ± 3.82 mm. The thickness values of the plates were generally thin (approximately 1.33–1.46 mm), with mean thicknesses of 1.46 ± 0.57 mm (right lateral), 1.42 ± 0.57 mm (left lateral), 1.34 ± 0.64 mm (right medial), and 1.33 ± 0.71 mm (left medial). The mean pterygoid angulation was approximately 50 to 52° bilaterally (51.91 ± 12.62° right; 49.92 ± 12.46° left). The average length of the styloid process was 22.99 ± 9.76 mm (males: 22.33 ± 9.79 mm; females: 23.97 ± 9.90 mm).

### 3.2. Sex Differences (Independent Samples t Test)

Regarding the comparisons between male and female participants, independent-samples *t*-test was computed. Our results revealed that the only statistically significant difference between males and females was in the length of the right lateral pterygoid plate, which was longer in males (15.74 ± 3.55 mm vs. 12.93 ± 3.31 mm; mean difference 2.81 mm, 95% CI 0.80–4.82; t (48) = 2.81, *p* = 0.007). All other variables exhibited no significant sex differences (all *p* > 0.05) (Table 2).

### 3.3. Correlation Analysis (Pearson’s r)

The Pearson correlation analysis demonstrated strong bilateral associations for key parameters. The right and left medial pterygoid plate lengths were strongly correlated (r = 0.729, *p* < 0.001), and right and left pterygoid angulation also presented a strong positive correlation (r = 0.632, *p* < 0.001), supporting substantial bilateral symmetry in these measurements. Additionally, a moderate negative correlation was observed between the length and thickness of the left medial pterygoid plate (r = −0.399, *p* = 0.004), indicating that longer left medial plates were generally thinner. The length of the styloid process did not exhibit any significant correlation with the lengths, thicknesses, or angulations of the pterygoid plates based on the correlation summary provided.

### 3.4. Side Differences (Paired t Tests)

Paired comparisons between pterygoid plates with respect to length, thickness and angle divergence is performed. (Table 3). The results show that there are no statistically significant differences between the right and left sides for lateral plate length (mean R–L 0.79 ± 4.46 mm, 95% CI −0.48 to 2.06; *p* = 0.218), lateral plate thickness (mean 0.04 ± 0.65 mm, 95% CI −0.14 to 0.22; *p* = 0.648), medial plate thickness (mean 0.01 ± 0.71 mm, 95% CI −0.19 to 0.21; *p* = 0.952), or pterygoid angulation (mean 1.99 ± 10.77°, 95% CI −1.07 to 5.05; *p* = 0.198). The RMP-LLP length was statistically significant (mean −2.56 ± 4.97 mm, 95% CI −3.97 to −1.15; *p* = 0.001); however, since it compares different structures (medial vs. lateral) rather than the same structure across sides, it should be regarded as a plate type difference rather than a side asymmetry.

### 3.5. Association with Age

In our entire sample (*n* = 50), age demonstrated no significant association with any morphometric parameter of the pterygoid plate (all |r| ≤ 0.155; all *p* ≥ 0.283) or the length of the styloid process (r = 0.093, *p* = 0.522). In sex-adjusted linear regression, age (per 10-year increment) revealed no association with pterygoid plate lengths, thicknesses, angulations, or styloid length (all *p* > 0.250) (Table 4).

## 4. Discussion

In our sample, the lengths of the lateral pterygoid plates (approximately 14–15 mm) and medial pterygoid plate (approximately 11–12 mm) provide specific evidence values for the adult population in Abha. In comparison to other studies, these dimensions are comparable to the ranges described in three-dimensional anatomical findings of the pterygoid plates and the posterior maxillary region, including the detailed morphologic characterization reported by Kwon et al. [2] Moreover, they emphasized that pterygoid plate morphology varies substantially among individuals and revealed a classification based on divergence where narrow-type morphology was found to be more prevalent than wide-type morphology. In Saudi populations, analyses based on CBCT have also demonstrated the feasibility of detailed morphometry related to the pterygoid region; however, comprehensive region-specific normative values remain limited, supporting the value of generating local reference data for Abha residents [15]. Therefore, our data contributes to region specific numerical reference values for the medial and lateral plates, as well as for plate angulation. Concerning sex-based variation, the primary observation was that males had a longer right lateral pterygoid plate compared to females (mean difference of 2.81 mm, 95% CI 0.80–4.82; *p* = 0.007). In contrast, other measurements such as lengths, thicknesses, angles, and the length of the styloid process did not show significant differences between sexes. This trend indicates that sexual dimorphism within this dataset is specific rather than universal across all posterior pterygoid metrics, which corresponds with previous findings. pattern in other Saudi populations, where males often demonstrate larger linear skeletal dimensions [16]. Given that numerous morphometric comparisons were conducted, this isolated significant result should be approached with suitable caution; nonetheless, the confidence interval that does not include zero suggests a probable genuine difference in right lateral plate length within this sample. In relation to the bilateral symmetry, the paired comparisons and correlation analysis indicate a significant agreement between the right and left sides in pterygoid morphology. The paired tests revealed no notable differences between sides for lateral plate length, lateral thickness, medial thickness, or pterygoid angulation (all *p* > 0.05, with confidence intervals including zero). Furthermore, strong positive correlations were identified between the right and left medial plate lengths (r = 0.729, *p* < 0.001) and between the right and left pterygoid angulations (r = 0.632, *p* < 0.001), which further underscores the symmetry of these measurements within the group. The statistically significant result regarding RMP-LLP length pertains to a comparison of plate types (medial versus lateral) rather than indicating true side-to-side asymmetry, aligning with the expected anatomical observation that lateral plates tend to be longer than medial plates. In the study reported by Kwon et al. [2], it was similarly noted that certain parameters varied between sexes (while many did not), supporting the concept that sex-related effects may exist but are selective rather than universal across posterior maxillary and pterygoid measurements. Conversely, Eberliköse et al. [1] reported higher average lateral pterygoid plate lengths in females than in males in a Turkish cohort assessed in the context of temporomandibular disorders, illustrating that sex-associated patterns may vary across populations and study contexts and may be influenced by differences in sampling frame, clinical characteristics, and measurement definitions. Concerning bilateral symmetry and internal morphology, the strong bilateral correlations observed for medial plate length and pterygoid angulation indicate a substantial symmetry in pterygoid plate morphology within this cohort. This aligns with prior anatomical work that reported minimal right–left differences in pterygoid plate dimensions and angles in general populations, including the lack of significant side differences reported by Kwon et al. [2]. Accordingly, the variability in morphometric studies of the pterygoid plate is affected by variations in the definitions of landmarks and the selection of planes. Recent research utilizing 3D/CT technology has established clear operational definitions for measuring plate length at specified levels and for assessing divergence angles, along with frameworks for morphological classification. By aligning measurements with these definitions, the comparability and interpretability across different populations are enhanced [2]. Additionally, the inverse association between left medial plate length and thickness suggests an internal morphologic relationship, wherein longer medial plates may present with reduced thickness. This finding may reflect biological variations in local craniofacial growth and remodeling, warranting further exploration in larger samples to determine whether this correlation is consistent and holds clinical significance for surgical risk assessment or implant planning. In relation to the length of the styloid process and its lack of association with the pterygoid plates, the average styloid process length in this cohort (23 mm) lies within the commonly cited normal range and remains below thresholds typically established for defining elongation. Reference [16] reported a normal styloid process length of approximately 20–30 mm, considering measurements that exceed 30 mm as indicative of elongation [18]. Previous studies have reported that the patterns of styloid process elongation and calcification vary by age and population [18,19,20]. In the summary of the current findings, styloid length did not exhibit significant correlations with the lengths, thicknesses, or angulations of the pterygoid plates, suggesting that variations in the stylohyoid complex may be largely operate independently of pterygoid plate morphometry within this population. From a clinical perspective, pterygoid plate morphology is highly relevant to posterior maxillary procedures—particularly controlled separation at the PMJ during Le Fort I osteotomy—and to implant rehabilitation strategies that engage posterior maxillary and pterygoid region bone. CBCT-based and radiologic investigations have linked regional bony anatomy and technique-related factors at the PMJ with fracture patterns and procedural safety considerations [8]. In addition, Limthanakul et al. [21] highlighted the importance of tailoring osteotome placement angles to pterygomaxillary anatomy during junction separation, supporting individualized preoperative assessment rather than relying on a single standard approach for all patients [19]. In the present cohort, mean pterygoid angulation was approximately 50 to 52°, with lateral plates exhibiting greater lengths than medial plates. The only significant sex effect observed was the increased length of the right lateral plate in males. When these results are analyzed in conjunction with previous morphological classifications regarding the divergence and variability of pterygoid plates, they further reinforce the clinical rationale for preoperative CBCT evaluation of plate length, thickness, and divergence [2]. Such evaluations are essential for guiding procedural trajectories and minimizing the risk of unwanted plate injury, consistent with contemporary recommendations in the surgical literature [8]. The current morphometric measurements can be considered beneficial in preoperative planning using CBCT for separating the pterygomaxillary region during Le Fort I osteotomy, as the anatomy of this area affects both fracture patterns and safety, and strategies for posterior maxillary rehabilitation that make use of anchorage in the pterygoid region. CBCT is frequently recommended as the preferred cross-sectional imaging method for evaluating implant sites when clinically warranted, with optimization principles underscoring the importance of indication-specific, patient-centered imaging. Future research that connects the morphometry of the pterygoid plate with surgical outcomes such as fracture patterns, bleeding, and primary stability of implants. Regarding limitations, the study population consisted of patients who received CBCT scans for clinical purposes instead of being sourced from a community cohort. Furthermore, measurements of extremely thin bony structures may be affected by partial volume effects as their dimensions near the voxel size. Additionally, our analysis predominantly utilized two-dimensional linear measurements on anatomically curved three-dimensional forms. Lastly, the sample size could restrict the accuracy of the estimated reference intervals, leading to greater uncertainty surrounding percentile-based normative values. In the context of pterygoid implant planning, the present morphometric values provide locally derived numeric constraints for posterior implant anchorage considerations and may be interpreted alongside three-dimensional studies of the pterygomaxillary region developed to support pterygoid implant approaches [9]. Considering our sample, it is crucial to establish population-appropriate morphometric reference values, as craniofacial measurements can differ across populations and may not be reliably extrapolated from external norms. CBCT-based studies in Saudi cohorts have demonstrated distinctive morphometric patterns in craniofacial structures, supporting the need for regionally grounded reference data [16]. By providing Abha-specific values for pterygoid plate length, thickness, angulation, and styloid process length, the present dataset offers clinicians a local benchmark for individualized planning and contextual interpretation of anatomical variability during posterior maxillary surgery and implant rehabilitation. The limitations of the current study pertain to its reliance on retrospective, clinic-based CBCT sampling; the scans were conducted for clinical purposes rather than for population screening that potentially restricting generalizability. Additionally, the measurements of the relatively thin plate may be influenced by partial-volume effects when cortical thickness nears the voxel size, as the data mostly consist of linear or angular descriptors rather than comprehensive 3D surface or volume segmentation. Overall, future multicenter studies involving diverse populations necessitate the establishment of age-stratified reference values and multivariable models (age, sex, dental status), the integration of skeletal patterns or craniometric classifications where the CBCT field-of-view permits reproducible 3D cephalometric landmarking, and the correlation of pterygoid plate morphometry with clinical outcomes including adverse pterygomaxillary fracture patterns, hemorrhage risk, primary stability of pterygoid implants.

## 5. Conclusions

In this retrospective CBCT study of 50 adults from Abha, the lateral pterygoid plates were consistently longer than the medial plates, and the pterygoid divergence angles were approximately 50° on both sides. Paired analyses revealed no significant differences between the right and left sides in lateral plate length, plate thicknesses, or pterygoid angulation, thereby affirming overall bilateral symmetry for these parameters. Sexual dimorphism was restricted to a longer right lateral plate in males, and the thickness of the left medial plate exhibited an inverse correlation with its length. Additionally, the length of the styloid process remained within anticipated parameters and demonstrated no significant relationship with pterygoid plate morphology. In total, these region-specific reference values may help with individualized preoperative CBCT assessment for planning posterior maxillary surgery and pterygoid implants.

## Figures and Tables

**Table 1:** Descriptive statistics for pterygoid plate and styloid measurements (mm or degrees).

Variables	Males Mean ± SD	Females Mean ± SD	Total Mean ± SD	Hedge’s g
Right lateral pterygoid length (mm)	15.74 ± 3.55	12.93 ± 3.31	14.61 ± 3.69	0.80
Right lateral pterygoid thickness (mm)	1.48 ± 0.59	1.42 ± 0.56	1.46 ± 0.57	0.10
Right medial pterygoid length (mm)	11.01 ± 3.38	11.66 ± 3.80	11.27 ± 3.52	−0.18
Right medial pterygoid thickness (mm)	1.29 ± 0.67	1.40 ± 0.61	1.34 ± 0.64	−0.17
Left lateral pterygoid length (mm)	13.62 ± 3.96	14.14 ± 3.99	13.83 ± 3.93	−0.13
Left lateral pterygoid thickness (mm)	1.44 ± 0.56	1.38 ± 0.62	1.42 ± 0.57	0.10
Left medial pterygoid length (mm)	12.20 ± 4.19	11.64 ± 3.27	11.98 ± 3.82	0.14
Left medial pterygoid thickness (mm)	1.41 ± 0.79	1.21 ± 0.59	1.33 ± 0.71	0.27
Right pterygoid angulation (°)	49.62 ± 13.49	55.36 ± 10.61	51.91 ± 12.62	−0.45
Left pterygoid angulation (°)	48.37 ± 13.89	52.25 ± 9.86	49.92 ± 12.46	−0.31
Styloid process length (mm)	22.33 ± 9.79	23.97 ± 9.90	22.99 ± 9.76	−0.16

**Table 2:** Sex-based differences of the pterygoid plates and styloid process morphometries.

Variable	t (df = 48)	Mean Diff (M–F) [95% CI]	*p*-Value
Right lateral pterygoid length	2.81	2.81 [0.80, 4.82]	0.007 *
Right lateral pterygoid thickness	0.38	0.06 [−0.28, 0.40]	0.705
Right medial pterygoid length	−0.63	−0.65 [−2.71, 1.41]	0.529
Right medial pterygoid thickness	−0.57	−0.11 [−0.49, 0.27]	0.571
Left lateral pterygoid length	−0.45	−0.52 [−2.83, 1.79]	0.655
Left lateral pterygoid thickness	0.41	0.06 [−0.28, 0.40]	0.686
Left medial pterygoid length	0.51	0.56 [−1.68, 2.80]	0.615
Left medial pterygoid thickness	1.01	0.20 [−0.22, 0.62]	0.320
Right pterygoid angulation	−1.60	−5.74 [−12.95, 1.47]	0.117
Left pterygoid angulation	−1.08	−3.88 [−11.11, 3.35]	0.286
Styloid process length	−0.58	−1.64 [−7.35, 4.07]	0.567

* Statistically significant.

**Table 3:** Pterygoid plates comparison in relation to length, thickness, and angle divergence.

(Right–Left) ^†^	Mean Diff ± SD	95% CI	t (df = 49)	*p*-Value
RLP–LLP length	0.79 ± 4.46	[−0.48, 2.06]	1.25	0.218
RLP–LLP thickness	0.04 ± 0.65	[−0.14, 0.22]	0.46	0.648
RMP–LMP thickness	0.01 ± 0.71	[−0.19, 0.21]	0.06	0.952
RIGHT–LEFT angle divergence	1.99 ± 10.77	[−1.07, 5.05]	1.31	0.198
RMP–LLP length	−2.56 ± 4.97	[−3.97, −1.15]	−3.64	0.001 *

* Statistically significant. ^†^ The comparison highlights the difference between structures (medial vs. lateral) rather than a right-left side difference.

**Table 4:** The association between the pterygoid plates morphometries with age.

Outcome	Pearson r	*p* (r)	β per 10 Years (adj. Sex)	95% CI	*p* (β)
Right lateral pterygoid length (mm)	0.151	0.296	0.23	−0.60 to 1.06	0.583
Left lateral pterygoid length (mm)	0.033	0.820	0.15	−0.80 to 1.11	0.746
Right medial pterygoid length (mm)	0.144	0.317	0.49	−0.35 to 1.33	0.250
Left medial pterygoid length (mm)	0.126	0.382	0.36	−0.56 to 1.28	0.435
Right lateral pterygoid thickness (mm)	0.021	0.885	0.00	−0.13 to 0.14	0.946
Left lateral pterygoid thickness (mm)	0.088	0.542	0.04	−0.10 to 0.18	0.594
Right medial pterygoid thickness (mm)	−0.155	0.283	−0.08	−0.22 to 0.07	0.302
Left medial pterygoid thickness (mm)	0.004	0.977	−0.02	−0.19 to 0.16	0.857
Right plate angulation (°)	0.039	0.789	0.91	−2.06 to 3.89	0.541
Left plate angulation (°)	−0.025	0.866	0.08	−2.91 to 3.07	0.958
Right lateral pterygoid length (mm)	0.093	0.522	0.91	−1.44 to 3.26	0.440

## Data Availability

The original contributions presented in this study are included in the article. For further inquiries, please contact the corresponding author.

## Abbreviations

The following abbreviations are used in this manuscript:

CBCT	Cone beam computed tomography
KKU	King Khalid University
CI	Confidence interval
PMJ	Pterygomaxillary junction
CT	Computed tomography
MDCT	Multidetector computed tomography
KKUCOD	King Khalid University College of Dentistry
